# A critique of financial neoliberalism: a perspective combining multidisciplinary methods and commodity markets

**DOI:** 10.1007/s43546-021-00054-9

**Published:** 2021-03-08

**Authors:** Pierluigi Vellucci

**Affiliations:** Department of Economics, Roma Tre University, Rome, Italy

**Keywords:** Financialization, Financial markets, Energy commodities, Futures, Multidisciplinary methods

## Abstract

The era of financialization bas been marked by a huge increase in the size of the financial sector. The economic justifications for this expansion arise from the prevailing neo-liberal ideology; they are based on arguments like leading role of markets, economic efficiency, reallocation and spreading of risk. Given these reasons, financialization can be viewed as a form of neoliberalism and then we can use the term *financial neoliberalism* (Palley in Financialization: the economics of finance capital domination. Springer, 2016) to denote it. This commentary paper hopes to complement this view by highlighting the usefulness of recent “multidisciplinary” methods (i.e., methods which do not fall within the usual economic discipline). We focus first on the definition of financialization process, second on its implications on the commodity markets, and third and finally on the importance of the role of multidisciplinary methods, applied to these markets, capable of highlighting the contradictions of the neoliberalism framework, in such a way, we hope, to promote further research.

## Rise and reasons of financial neoliberalism

The era of financialization has been marked by a huge increase in the size of the financial sector. To have an idea about this growth, it is sufficient to mention the tremendous rise of the ratio between financial and domestic corporate profits (Di Bucchianico 2020a, b) or the flows into commodity investments, increased from $ 15 billion in 2003 to $ 250 billion in 2009 (Irwin and Sanders 2012). Financial institutions, insurance companies, pension funds, among others, can be considered the major causes of these vast inflows (Zhang et al. 2017; Wang et al. 2015; Mi et al. 2017; Benedetto et al. 2019). According to the US government, the share of US GDP produced by finance, insurance and real-estate industries has risen from 15 to 24 per cent making it bigger than manufacturing and close to the size of the service sector (Mason 2016; Krippner 2005). Dávila-Fernández e Punzo (2020), focussing on the United States for periods 1947–2015, find that the service industries exhibit an increased importance of the financial sector after the 1980s. Similar results are found also in EU. Spanò (2019), measuring financial flows within the 14 founder countries of the European Union (EU14), on a sector-by-sector basis, over 21 years (1995–2015), shows that an increasing amount of credit in the EU14 group of countries has involved transactions of assets already in place (housing, stock market) or of newly generated financial assets, revealing that a prevalent and growing share of the gross excess finance has circulated within the domestic and foreign financial sector only. In other words, without contributing to the generation of real income.

The economic justifications for this expansion arise from the prevailing neo-liberal ideology; they are based on arguments like these:

- Markets coordinate economic activity in an optimal fashion: they should be deregulated or created if possible. According to this view, the market is not an economic but a political project; it entails the reengineering of social organization and coordination (and not their dismantling). In this context, financial markets are held up as the ideal market because it is expected that financial prices can embody all economically relevant available information.
- Financial markets play a special economic role regarding the allocation of saving. They perform the role of financial intermediaries (banks and the loanable funds market), by channelling funds from surplus economic units (savers) to deficit units (borrowers). Financial intermediation therefore ensures full employment. It also increases growth by allocating saving to those who require liquid funds to carry out a desired activity and generate the highest returns. And the higher returns earned from lending make saving more attractive. As a result, financial intermediation also increases investment and saving.
- According to the efficient market approach, financial markets increase capital accumulation and income. This is through creation of liquid asset markets in which firms are correctly evaluated according to their “fundamentals”, that is, their potential profitability (Ghosh et al. 2012). This means that assets are efficiently priced since liquid financial markets are engaged in information processing and price discovery activities. The information processing leads, on the one hand, to firms to operate more efficiently and, on the other, to capital market traders to be rewarded (on the contrary punished) for trading at prices that reflect (on the contrary misread) fundamentals. As a result, economic agents can direct their income to the accumulation of productive assets that raise income and growth (Palley, 2016).
- The reallocation and spreading of risk represent another function of financial markets, which can be done through insurances. They were mainly employed by insurance companies to protect themselves against future catastrophic losses, and in the past decades, they have been extended by futures contracts, whose original use was to mitigate the risk of price or exchange rate movements. Risk management allows in turn producers to be more willing to undertake risky productive activity.

Given these reasons, financialization can be viewed as a form of neoliberalism and then we can use the term *financial neoliberalism* (Palley 2016) to denote it, which is characterized by domination of the macro economy and economic policy by financial sector interests. This means that financialization phenomenon cannot be understood without an understanding of the neoliberalism in which it is naturally embedded.

As a starting point for our analysis of financialization phenomenon, we have considered the price linkages between financial indexes and commodity prices (e.g., for energy and food) which are able to reveal an increase in financial content underlying the process of commodity prices forming. In fact, although commodity derivatives were initially designed for protecting investors against losses that may result from adverse market price fluctuations on the underlying commodity, they are also widely used by speculators (Girardi 2012).

This commentary paper hopes to complement this view by highlighting the usefulness of recent “multidisciplinary” methods (i.e., methods which do not fall within the usual economic discipline and which some experts may not recognize as relevant in it). We focus first on the definition of financialization process, second on its implications on the commodity markets, and third and finally on the importance of the role of multidisciplinary methods, applied to these markets, capable of highlighting the contradictions of the neoliberalism framework, in such a way that, we hope, to promote further research.

## Socio-economic implications of financial neoliberalism

Since the 1980s, banks turned to consumers as a new source of profit, and to a set of high-risk, complex activities that we call investment banking (Mason 2016). Since then, credit cards, mortgages, overdrafts, student loans and motor car loans have become part of everyday life and thus consumers became direct participants in the financial markets. A growing proportion of profit in the economy is now being made by lending money to workers. Nevertheless, while the stagnation of real wages is lasting, a result of financial neoliberalism is to change in the income distribution. To explain this, we will use the symbols without (resp. with) asterisk to denote the variables before (resp. after) financialization process. Observe that GDP can be decomposed into capital’s (C) and labour’s share (L); then, $${\text{GDP}} = C + L $$, before financialization, and $${\text{GDP}}^{*} = C^{*} + L^{*}$$ after financialization. Hence, financial neoliberalism has been such that $$C^{*} > C$$ and $$L^{*} < L$$. Labor’s share can in turn be decomposed into managers’ share (M) and non-managers’ share (NM); with the previous notation: $$L = M + NM$$ and $$L^{*} = M^{*} + NM^{*}$$. Financial neoliberalism has been such that $$M^{*} > M$$ and $$NM^{*} < NM$$. Capital’s share can be broken down into profits (P) and interest income (I), and profits is such that $$P = F + NF$$, where is the financial sector profits while NF is the non-financial sector profits. The era of financialization has also seen $$I^{*} > I$$ and $$P^{*} < P$$ (Palley 2016; Mishel et al. 2009). On the other hand, the rise of financial neoliberalism has seen the increase in the indebtedness of households and businesses. See, e.g., the burgeoning household debt in percentage of GDP in the US (Di Bucchianico 2020a, b); or the growing of subprime lending i.e., mortgages for customers not deserving of creditworthiness, in other words “poor people”, at high real interest rates from $160 billion to $600 billion (Lapavitsas 2009). This represent an unprecedented occurrence in the history of capitalism: in fact, from the above considerations, if a declining share of money flows to workers, and yet a growing part of profits is generated out of their mortgages and credit cards, the music will stop, in terms of liquidity, and the things will be complicated (paraphrasing the words of the former Citigroup CEO Chuck Prince). That is exactly what happened when the US subprime mortgage bubble collapsed.

In Sect. 1, we mentioned the reallocation and spreading of risk among the economic justifications that neo-liberal ideology adduces for the expansion of financial neoliberalism. But we should first distinguish between two “parts” of finance: business and speculation. The first promotes healthy development of capitalist economies and is important for long-term economic development; the second, instead, can lead to explosive behaviour and the bursting of bubbles, with consequent negative economic consequences. For the latter, just think of the US subprime mortgage bubble collapsed: issuing credit to borrowers with poor creditworthiness profiles does not reduce risk, instead it favours its spreading. Not to mention the widespread trading of derivatives has not always been linked to the desire to buy insurance, but rather to simply engaging in activities that recall a bet at the casino or racetrack.

As finance has entered the daily life of workers, they not only generate profits for their bosses through their work, but also profits for financial intermediaries through their loans. In fact, as written by Mason (2016), “a single mum on benefits, forced into the world of payday loans and buying household goods on credit, can be generating a much higher profit rate for capital than an auto industry worker with a steady job.”

After 1980, the Keynesian growth model of the virtuous circle was replaced by the neo-liberal growth model, which involved, inter alia, breaking the link between wages and productivity growth. The new growth model has made credit and (low) asset price inflation the engines of demand growth. Dependence on debt and asset price inflation has thus placed the financial markets at the centre of the economic process, ultimately weakening the position of workers. Hence the paradigm of financial neoliberalism.

The neo-liberal model created a growing structural gap in aggregate demand, and the role of finance was to bridge that gap. After suffering the blows of the 2008 financial crisis, politicians managed to stabilize the system and prevent a second Great Depression. The fiscal stimulus packages of 2009 strengthened aggregate demand. The bailout of the banks in 2008–2009 and the provision of emergency liquidity halted the flight from financial assets that had threatened to bankrupt the system (Palley 2016; Mason 2016).

These measures stabilized the system but did not reform the structure of the economy. Furthermore, the economic system is still burdened by the structural demand gap caused by the deterioration in income distribution. Moreover, the recent pandemic of coronavirus disease 2019 (COVID‐19) has further aggravated these problems and forced countries to deal with a deep economic stagnation (which, however, was clear even before the pandemic, as a side effect of the neo-liberal economic system).

## Evidence of financialization on commodity markets

Price linkages between financial indexes and commodity prices (e.g., for energy and food) can be seen as a footprint of the financialization phenomenon, since they reveal an increase in financial content underlying the process of commodity prices forming. The studies focussing on these relations have attracted widespread attention, especially upon the occurrence of concurrent swings in agricultural commodity and crude oil prices.

According to Girardi (2015), a combination of financialization and financial crisis seems to drive the increasing correlation between agricultural prices and stock market dynamics. It is therefore likely that the influence of financial shocks on agricultural prices will diminish as global financial tensions fade. But the flip side is that, if agricultural derivatives markets are populated mainly by financial investors, it can be expected to rise again in the presence of new financial turmoil. Although commodity derivatives were initially designed for protecting investors against losses that may result from adverse market price fluctuations on the underlying commodity, they are also widely used by speculators (Girardi, 2012). Tang and Xiong (2012) claim that, since the early 2000s, prices of non-energy commodity futures in the United States have become increasingly correlated with oil prices and this trend reflects the financialization of the commodity markets and helps explain the large increase in the price volatility of non-energy commodities around 2008. Zhang et al. (2017), Benedetto et al. (2019) investigate the role of equity markets in relation to crude oil and natural gas markets, showing that de-financialization for crude oil and natural gas markets after 2008 is not detectable in the data.

## The need of multidisciplinary methods

Conventional economic theory argues that the expansion of financial markets enhances economic efficiency (Palley 2016). Economic efficiency can be characterized in many ways; one of the best known in finance is the efficient-market hypothesis (EMH). The efficient market hypothesis is associated with the idea of a “random walk” which is a term loosely used in the finance literature to characterize a price series where all subsequent price changes represent random departures from previous prices: “The logic of the random walk idea is that if the flow of information is unimpeded and information is immediately reflected in stock prices, then tomorrow's price change will reflect only tomorrow's news and will be independent of the price changes today. But news is by definition unpredictable, and, thus, resulting price changes must be unpredictable and random. As a result, prices fully reflect all known information, and even uninformed investors buying a diversified portfolio at the tableau of prices given by the market will obtain a rate of return as generous as that achieved by the experts” (Malkiel 2003). This is the weak form efficiency, also known as the random walk theory, according to which future prices are random and past information has no relationship with current market prices.

But, at least looking at the commodity futures price series, we are not sure that economic efficiency is improved by the expansion of financial markets. We refer to Mastroeni et al. (2018, 2019); these papers address the predictability of copper and energy futures markets, by employing “multidisciplinary” methods, i.e., a methodological approach which is not part of the economic discipline and then it cannot be considered within the set of “mainstream” tools. This methodological approach is taken from Physics, Mathematical Physics or Information Theory and rely on the concept of dynamical system.

What we have defined as “multidisciplinary methods” are, by definition, across several fields of study and then come out of the widely accepted approaches in economics, that usually derive from statistics and econometrics. However, there are profound differences between these tools and those proposed in this article. There is no better or worse, but we believe that the accepted approaches in economics may benefit from the integration with multidisciplinary tools. For example, as pointed out by Benedetto et al. (2019, 2020) or Mastroeni et al. (2018, 2019), while many well-established approaches in economics usually require parametric models, the multidisciplinary methods we propose in this commentary paper are mainly non-parametric methodologies, which do not require to specify a functional form for the relationships among variables.

The point is the following: if the expansion of financial markets enhances economic efficiency, and if the prices are truly random because governed by a random walk (then stochastic) process, why Mastroeni et al. (2018, 2019) found that the time series of logarithmic returns has both a stochastic and a deterministic nature? Mastroeni et al. (2019) can estimate how far the system underlying the price formation of natural gas, crude and heating oil, is from a purely stochastic behaviour. It is achieved through a measure for determinism of the system, denoted by DET (Marwan et al. 2007). The so-called determinism coefficient DET arises from the fields of descriptive statistics and chaos theory, and it is based on the concept of *recurrence plot*, a plot that accounts for returns of the trajectory in the state space to a neighbourhood of a point crossed in the past (recurrence points). DET is defined as the percentage of recurrence points that form diagonal lines with minimum length. This percentage is equal to one for a deterministic system, while DET = 0 for a random walk. This would be important from a policy point of view if, hypothetically, it was possible to clearly separate stochastic and deterministic components of energy time series. In fact, while the behaviour of purely stochastic trajectories is hard to be predicted anyway, it is possible to predict a deterministic system on short periods of time. For example, some of the price time series considered by Mastroeni et al. (2019) show a determinism coefficient DET near to 0.9. In other words, for these commodities, this approach suggests that there should be some deterministic forces interacting with each other in such a way that complicated price movements can be produced in their markets. A mention to these “deterministic forces” can be found also in Panas and Ninni (2000). After all, risk-neutral speculators who trade solely based on noise can generate market inefficiency if other operators are risk averse (De Long et al. 1990). We believe this is a point to be addressed in future studies.

If we remain within the neo-liberal theoretical framework described so far, we could object that values of DET so close to 1 could be due to the phenomenon of financialization, which may not be as evident as claimed in the literature. The financialization process is instead present, as we can infer from another multidisciplinary tool, which is taken from Information Theory. It is the concept of Shannon Entropy introduced by Shannon (1948) the “father of information theory” and provides a way to estimate the average minimum number of bits needed to encode a string of symbols (emitted by a source X), based on the frequencies of the symbols. In the definition of Shannon, given a source *X* that can transmit* N* symbols (where each *i*-th symbol is characterized by an a-priori probability $p_{i}$), the entropy is defined as:

$$ E\left( X \right) = - \mathop \sum \limits_{i = 0}^{N - 1} p_{i} \cdot \log_{2} p_{i} $$

Symbols which are more probable than others produce observations (i.e., receptions) which are less informative for the receiver. Conversely, rarer symbols provide more information when observed. The entropy is zero when an outcome is certain. In practical terms, the information is the removal of the uncertainty: high values of the Shannon entropy result in an unpredictable series, while lower values mean less uncertainty and hence a more predictable behaviour of that series. Thanks to this concept, Benedetto et al. (2019) prove that the financialization of energy markets remains after the 2008 financial crisis. They model how the information content, quantified by Shannon entropy, flows from one series to the other one. With similar entropy-based concepts, Benedetto et al. (2020) examine the flow of information and its direction between the oil volatility index (OVX) and the spot variance of WTI and Brent returns. Their results show that the bulk of information flow comes from OVX to the spot variance of the two oils. They write that, “one of the possible explanations of this significant information transfer may be the process of financialization of the oil market. Financialization of the oil market caused a growth in the number of investors trading in oil derivatives and on commodity ETF and may have strengthened the linkages between the crude oil spot market and the financial one.”

These approaches have the advantage that they do not require a parametric model describing the transmission of information between the time series under investigation.

The expansion of financial markets has been promoted not only by appeal to the theory of efficient markets (Fama 1970), but also by claiming that speculation is stabilizing (Friedman 1953; Palley 2016). Nevertheless, there is a literature that challenges the latter conclusion. According to rational expectations theory, market participants can rationally participate in bubbles if they have expectations of price increases, i.e., it stays rational for investors to remain in the market provided they are compensated by a higher rate of growth of the bubble for taking the risk of a crash (Flood and Garber 1980; Sornette 2003). Recent literature has introduced bubble detection methodology based on the Log Periodic Power Law Singularity (LPPLS), which has been used to detect bubbles in many contexts (Zhang et al. 2016): it seems that speculation is not stabilizing. LPPLS differs from other methods (Phillips 2015) because its methodological approach does not require econometric modelling and therefore can be considered in all respects a “multidisciplinary” tool (Benth et al. 2013; Fantazzini 2019). On the same class of methods, but based instead on the entropy concept, are those introduced by Stosic et al. (2016) and Bhaduri (2014). Empirical results suggest that financial crises are associated with significant increase of exchange rate entropy (Stosic et al. 2016), reflecting instability in FX market dynamics, and that there are strong “tell-tale” signs characterized by low Approximate Entropy (ApEn) level during many of crash events (Bhaduri 2014). The approaches followed by Stosic et al. (2016) and Bhaduri (2014) have the advantage that, as is the case for the entropy-based tools, they do not require a parametric model and work directly on the data.

Speculation. It means speculators. According to an established literature, the speculators should increase the efficiency in the future markets (Buyuksahin and Harris 2011; Bekaert and Harvey 2000). Nevertheless, the academic literature has so far not settled on the question whether macroeconomic fundamentals or instead speculators were mainly responsible for the strong fluctuations in commodity prices (Bohl et al. 2019). As Bohl et al. (2019) have pointed out in their literature review, “little is known about the influence of speculative trading on the efficiency of commodity futures markets”. Such influence could lead to an improvement or a worsening of market efficiency. In fact, if on the one hand the speculators that primarily act on proprietary information should facilitate market efficiency, as well as a higher market liquidity, on the other, the order flow by poorly informed noise traders could prevent the information transmission (Bohl et al. 2019).

The last point we would like to address is herding behaviour. It is an important feature of financial market behaviour that can promote financial instability; the assumption is that agents tend to imitate the opinions of their “neighbours”, not contradict them (Sornette 2003). The key argument of this view is that it is optimal to imitate when lacking information. Traders are indeed constantly sharing information, calling each other to “take the temperature”, effectively polling each other before taking actions. This is related to the socially propelled conventions analysed by Keynes (1930, 1936, 1937); in times of uncertainty, these conventions encourage speculators to believe what others believe and to do what others do. Herding behaviour is an interesting microeconomic behaviour; but above all, it is an important mechanism for providing micro foundations to Minsky’s (1982) financial instability hypothesis, as examined by Palley (2016). The challenge is however to explain herding behaviour in a way that is coherent with rational individual decision-making. Herding behaviour is consistent with rational individual decision-making since the actions carried out by agents convey valuable information about their private decision making. The formalism of Agent-Based Model (ABM), which can be used to model micro and macro phenomena, represent a useful tool to investigate herding behaviour. Several experimental works on group psychology has been done in recent years to observe unexpected dysfunctional behaviours in decision-making communities (Pareschi et al. 2017; Mastroeni et al. 2020); as a future perspective, it can be interesting to include them in a rational individual decision-making model for herding behaviour.

## Conclusions

In this work, we advocate the use of multidisciplinary methods to study economic and social phenomena and processes such as financialization, speculation, herding. Why do we decide to use the examples of multidisciplinary methods, instead of using widely accepted approaches? What we have defined as "multidisciplinary methods" are, by definition, across several fields of study and then come out of established approaches in economics, that usually derive from statistics and econometrics. However, there are profound differences between these tools and those proposed in this article. There is no better or worse, but we believe that the accepted approaches in economics may benefit from the integration with multidisciplinary tools. For example, the multidisciplinary methods we propose in this commentary paper are mainly non-parametric methodologies, which do not require to specify a functional form for the relationships among variables.

The results obtained using these tools, which in other disciplines have produced robust and widely accepted findings for decades, cannot, in my opinion, be labelled as marginal in the economic academic community but require thorough review even when they seem to refute some mainstream economic theories. Then it seems that some of the economic justifications that led the expansion of financial sector are at least controversial, as discussed in previous sections. But it is not the purpose of this commentary paper to put an end to the debate on the financialization of commodity markets and financial capitalism. The goal was instead to underline the importance of the role of multidisciplinary methods, applied to these markets, capable of highlighting the contradictions of the neoliberalism framework, in such a way that, we hope, to promote further research.

## Data Availability

Not applicable.
